# Organophosphate-Pesticide-Mediated Immune Response Modulation in Invertebrates and Vertebrates

**DOI:** 10.3390/ijms24065360

**Published:** 2023-03-10

**Authors:** Karime Guadalupe Bernal-González, Carlos Eduardo Covantes-Rosales, Milton Rafael Camacho-Pérez, Ulises Mercado-Salgado, Victor Wagner Barajas-Carrillo, Daniel Alberto Girón-Pérez, Ashley Carolina Montoya-Hidalgo, Karina Janice Guadalupe Díaz-Resendiz, Rocío Guadalupe Barcelos-García, Gladys Alejandra Toledo-Ibarra, Manuel Iván Girón-Pérez

**Affiliations:** 1 Maestría en Ciencias Biológico Agropecuarias, Universidad Autónoma de Nayarit, Xalisco 63780, Nayarit, Mexico; 2 Laboratorio Nacional de Investigación para la Inocuidad Alimentaria (LANIIA)-Unidad Nayarit, Universidad Autónoma de Nayarit, Tepic 63173, Nayarit, Mexico; 3 Doctorado en Ciencias Biológico Agropecuarias, Universidad Autónoma de Nayarit, Xalisco 63780, Nayarit, Mexico; 4 Licenciatura en Biología, Universidad Autónoma de Nayarit, Xalisco 63780, Nayarit, Mexico

**Keywords:** organophosphorus pesticides, diseases, immune system, immunotoxicity, infections, non-target organisms

## Abstract

Organophosphate pesticides (OPs) have greatly facilitated food production worldwide, and their use is not limited to agriculture and the control of pests and disease vectors. However, these substances can directly affect the immune response of non-target organisms. In this sense, exposure to OPs can have negative effects on innate and adaptive immunity, promoting deregulation in humoral and cellular processes such as phagocytosis, cytokine expression, antibody production, cell proliferation, and differentiation, which are crucial mechanisms for host defense against external agents. This review focuses on the scientific evidence of exposure to OPs and their toxic effects on the immune system of non-target organisms (invertebrates and vertebrates) from a descriptive perspective of the immuno-toxic mechanisms associated with susceptibility to the development of bacterial, viral, and fungal infectious diseases. During the exhaustive review, we found that there is an important gap in the study of non-target organisms, examples of which are echinoderms and chondrichthyans. It is therefore important to increase the number of studies on other species directly or indirectly affected by Ops, to assess the degree of impact at the individual level and how this affects higher levels, such as populations and ecosystems.

## 1. Introduction

Pesticides are substances widely used in the field of agriculture and health for the elimination of food pests, reduction of food losses, and control of vectors transmitting human and animal diseases [1,2]. Currently, due to the increase in the world population and its need to produce more food, as well as to avoid losses in agricultural crops, the use of pesticides has increased. Organophosphates pesticides (OPs) belong to the group of the most widely used pesticides, which are derived from phosphorous compounds, such as phosphoric and phosphorothioic acid. Globally, about 2 million tons of pesticides are used every year; however, as of 2020, the annual increase of these substances was estimated at 3.5 million tons, of which approximately 40% represent OPs [1,3]. Additionally, an estimate of more than 3 million people are exposed to OPs each year, leading to 300,000 deaths worldwide [4].

Although OPs have been used to control pests for more than 50 years [5], the use of OPs has increased considerably since the banning of organochlorine pesticides, because OPs have limited persistence in the environment and pose a lower risk to human health compared to organochlorines; however, excessive use, poor storage, transport, application, and disposal of residues pose a latent risk of affecting non-target organisms [6,7,8]. OPs are incorporated into organisms by three routes of exposure: oral, respiratory, and dermal. In terms of absorption, the main absorption route is through the diet, while respiratory absorption will depend on physicochemical properties and environmental persistence. On the other hand, dermal absorption is influenced by polarity and solubility. Once absorbed, the compound is distributed in the organism, and subsequently bio-transformed to more hydrophilic metabolites to increase polarity and facilitate elimination. The main organ where biotransformation takes place is the liver, although this phenomenon also occurs in the kidney, intestine, and gills [9,10]. In vertebrates, biotransformation is a process that takes place in different stages, involving both bioactivation and detoxification [11]. OPs are mainly bio-transformed in the liver via CYP450 (phase I) by chemical reactions (oxidative desulfurization), to form highly toxic compounds, such as oxon metabolites. The metabolic process of detoxification consists of reactions (phase II) of dearylation and hydrolysis to convert the oxon into dialkyl phosphates (DAPs), dialkyl thiophosphates (DATPs), and 2-isopropyl-4-methyl-6-hydroxy pyrimidine (IMPH), which are highly soluble metabolites that can be excreted mainly through urinary conjugation reactions [7,8,12,13].

OPs have different mechanisms of action, the most studied being the neurotoxic mechanism, which consists of the inhibition of the enzyme acetylcholinesterase (AChE), by phosphorylation of the amino acid serine at its carboxyl-terminal residue present in the active site of the enzyme. This inhibition leads to an inability to hydrolyze the neurotransmitter acetylcholine (ACh), increasing its levels at the nerve synapse, resulting in the overstimulation of muscarinic and nicotinic receptors, which could cause both neuronal and non-neuronal toxic effects in exposed organisms [14,15]. Furthermore, it has been reported that oxons can directly interact with muscarinic receptors, such as M2 and M4, and dysregulate cAMP-mediated cell signaling processes [16,17,18]. It has also been described that secondary metabolites, such as diethyl thiophosphate (DEPT), alter the lymphocyte response by modulating IL-2 receptor-mediated signaling [19]. In this sense, the dysregulation of the cholinergic system by OPs could be related to sensory, motor, immunological, endocrine, and neurological alterations, leading to the development of various pathologies, such as cancer, hypersensitivity, neurodegenerative diseases, infections, and diabetes [20,21,22,23].

**Figure 1:**
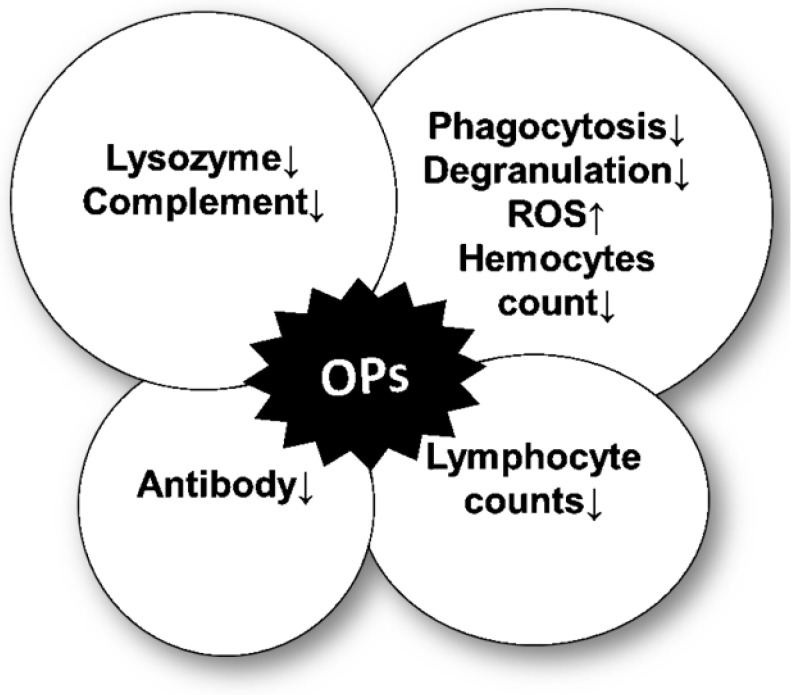
Main alterations of humoral and cellular mechanisms of innate and adaptive immunity mediated by OP exposure (Increase ↑, decrease ↓).

Scientific evidence of in vivo and in vitro assays demonstrated the toxic effect of OPs on the immune response of several organisms (Figure 1), but the non-neuronal molecular mechanisms of these effects are not fully elucidated. In this sense, it is suggested that the reported effects could be related to interactions of active metabolites of OPs on nicotinic, G-protein-coupled muscarinic (GPCR) and interleukin (ILR) receptors, altering the signaling pathways and gene expression in cells of innate and adaptive immunity, thereby modulating processes of phagocytosis, respiratory burst, lymphoproliferation, cellular senescence, neutrophil extracellular traps (NETs), cell death, complement, antibody production, cytokines, chemokines, and antigen presentation [18,19,24,25,26,27,28,29,30,31,32].

## 2. Immune Response: Evolutionary Overview

Life began on our planet more than 3.5 billion years ago, and evolving single-cell organisms, archaea, bacteria, and eukaryotes, have flourished ever since. Around 600 million years ago, multicellular organisms (metazoans) began to form in conjunction with a dramatic increase in atmospheric oxygen levels. This development was followed by a remarkable diversification of metazoan species in such a relatively short period that has been called the “evolutionary big bang.” [33]. The evolutionary emergence of vertebrates was accompanied by major morphological and functional innovations, including the development of an immune system [34].

Innate immunity is the first line of defense against antigens [35]. Innate mechanisms are the first to respond to an antigen, recognizing it in a generic and non-specific way, thus generating a rapid response to eliminate it, but it does not provide long-term protection [36,37,38].

Innate immunity includes effector molecules, such as interferons, complement proteins, natural antibodies, growth inhibitors, and protease inhibitors, and cells such as macrophages, monocytes, neutrophils, and mast cells. Additionally, included are species-specific physical barriers such as mucus, skin, gills, intestines, and nostrils [37]; whereas, the cellular defense of invertebrates is carried out by hemocytes through phagocytosis, cytotoxic reactions that include the release of lysosomal enzymes and antimicrobial peptides, and respiratory burst [39].

Innate immunity is considered evolutionarily older than adaptive immunity [35]. Even unicellular organisms have heritable defense mechanisms, and every multicellular organism appears to have a complex innate immune system [40]. The basic protective strategy of an innate immune system is for the organism to constitutively produce generic receptors that recognize conserved patterns on different classes of pathogens to trigger an inflammatory response that limits pathogen invasion [33]. Innate immunity is of paramount importance for both invertebrates and some lower vertebrates, such as fish [38], to the extent that it has been suggested that teleost fish have a more robust innate response than mammals [35]. In addition, they have an instructive role for adaptive immunity mechanisms [38].

Jawed vertebrates (gnathostomes) possess a remarkably adaptive immune system that can recognize and initiate a protective response against potentially lethal pathogens, including bacteria, viruses, fungi, and parasites [33]. In this way, the adaptive immune system responds specifically against the antigen that triggered the immune response [37]. Specific adaptive immunity depends upon the somatic diversification of antigen receptor genes to generate a vast repertoire of cells, each of which expresses a different antigen receptor [33].

Adaptive immunity is composed of highly specialized cells, which include populations of lymphocytes, such as B cells, T cells, and natural killer (NK) cells, as well as immunoglobulins and the major histocompatibility complex (MHC) system [35,36,37]. Lymphocytes, the specialized cell type of the adaptive immune system, use their cell-surface receptors to recognize antigenic configurations of specific pathogens and then respond to the antigen triggering by clonal amplification, cellular differentiation, and antibody production with the same antigen-binding specificity [33]. The TCRs recognize peptide fragments of antigens presented by other cells within cell-surface molecules encoded by the major histocompatibility complex (MHC) class I and class II genes. T cells, therefore, typically recognize antigens that have been partially digested by the antigen-presenting cells, primarily dendritic cells, phagocytic cells, and B cells. The membrane-bound and secreted antibodies made by B lineage cells, by contrast, recognize exposed determinants (epitopes) of intact molecules, including surface protein and carbohydrate moieties of invasive microbes [33]. Even cartilaginous fish, such as sharks, have TCR and BCR genes divided into V, D, J, and constant I region segments and *RAG1/RAG2*, *MHC I*, and *MHC II* genes [33].

A key feature of adaptive immunity is the development of immunological memory, as memory cells are generated that provide long-lasting specific immunity, and thus play a crucial role in protecting against recurrent infections with a rapid, intense, and efficient response [36,37,38], leading to an appropriate response in subsequent encounters with the antigen [41]. Interestingly, memory formation, previously thought to be a defining feature of adaptive immunity, also occurs in the context of innate immune responses and can be observed even in unicellular organisms, demonstrating the convergent evolutionary history of different aspects of adaptive immunity [34].

## 3. OP-Mediated Modulation of the Immune Response

### 3.1. OP-Mediated Modulation of the Innate Immune Response in Invertebrates

OPs are also able to decrease innate functions of hemocytes in invertebrates. Notwithstanding the above, OPs dysregulate immune functions, and in line with this, the hemocyte count is altered in *Drosophila melanogaster* Meigen exposed to acephate (acute and chronic) [42,43,44]. Contrastingly, monocrotophos, dimethoate, and methyl parathion increased the total hemocyte count in *Rhynocoris kumarii* Ambrose and Livingstone [45]. Moreover, hemocyte count alterations have been reported in shrimps (*Litopenaeus vannamei* Boone) exposed to malathion [46]. In addition, modifications in hemocyte counts were reported in calico (*Porcellio scaber*), a medium-sized terrestrial isopod [47]. *Philosamia ricini* (eri silkworm) exposed to chlorpyrifos deregulated phenoloxidase and lysozyme response, as well as the hemocyte count [48].

In this regard, azamethiphos and dimethoate decreased the phagocytic index in the marine mollusk *Mytilus edulis* L. [49,50]. Alongside this, in gastropods *Planorbarius corneus* L. and *Biomphalaria glabrata* Say, exposed to chlorpyrifos, the hemocyte count and lysozyme activity were not significantly altered; notwithstanding, functional parameters (phagocytosis and ROS production) were altered [39]. Futher, hemocyte viability and phagocytic activity decreased after azinphos-methyl exposure in the freshwater snail *Chilina gibbosa* G. B. Sowerby I. [51]. In the mussel *Diplodon chilensis* Gray, the exposure to azinphos-methyl modified the hemocyte count, enzymatic activity (lysozyme and phenoloxidase, and glutathione S-transferase), and phagocytic activity [52]. Additionally, in *Mytilus galloprovincialis* exposed to chlorpyrifos, parameters of the immune response, lysosomal membrane stability (LMS), reactive oxygen species (ROS) production, and DNA damage were altered in hemolymph and hemocytes [53].

Furthermore, alterations in cellular functionality have also been reported in crustacean (such as the American lobster (*Homarus americanus* H. Milne-Edwards)) sub-lethal exposure to malathion, where phagocytosis was altered [54]. Meanwhile, in the giant freshwater prawn (*Macrobrachium rosenbergii* De Man), trichlorfon exposure decreases the hemocyte phenoloxidase activity and respiratory burst [55]. Similar results were reported by [56], who also observed alterations in enzymatic activity (phenoloxidase and superoxide dismutase (SOD)), as well as gene expression (prophenoloxidase, the lipopolysaccharide and β-1,3-glucan-binding protein, peroxinectin, α2-macroglobulin, transglutaminase, and copper, zinc (Cu, Zn)-SOD) [56]. While in shrimp (*Penaeus monodon*), genotoxicity has been seen in the hemocytes of organisms exposed to malathion and monocrotophos [57].

Gene expression analysis in response to chlorpyrifos and diazinon in *Caenorhabditis elegans Maupas* (*C. elegans*) revealed alterations in transcriptional response related to innate immunity (lysozyme and saposin downregulation) [58,59]. Hence, environmental exposure to OPs may result in harmful invertebrate immunity, leading to susceptibility to infections.

### 3.2. OP-Mediated Modulation of the Innate Immune Response of Vertebrates

No target species (wildlife) populations have been threatened in diverse geographic locations. Multiple causes have been suggested to explain this decline. Environmental pollution may explain such affectations. Indeed, some chemicals of environmental concern were reported to alter the immune system [60]. Although several authors have reported acute adverse effects caused by OPs on non-target wildlife species (invertebrates and vertebrates, such as fish, amphibia, birds, reptilia, and mammals), however, little attention has been given to the immunotoxicity effects of long-term exposure [26].

#### 3.2.1. Fish

To date, information on the immuno-toxic effect of OPs on agnathans and Chondrichthyes is scarce. Fish have been used as indicator species for water quality contamination, because fish are organisms closely related to the aquatic environment and suffer stress and disturbance due to the presence of pollutants [61]. Therefore, organisms such as medaka, zebrafish, carp, guppy, and tilapia are often used as models for toxicological testing [62].

Studies have focused on the toxic effect on bony fish. OPs induce changes in fish immune cell counts, as has been observed in invertebrates. In this regard, white blood cell (WBC) counts increased in common carp (*Cyprinus carpio carpio* L.) exposed to chlorpyrifos [63]. Alongside that, other immune cell counts can be modulated, as reported in *Oncorhynchus mykiss* Walbaum exposed to diazinon, where monocytes were lower, while a significant increase in neutrophils was observed [64]. In line with this, neutrophilia induced by diazinon was also described in *Pangasius hypophthalmus* Sauvage [65]. Hematological parameters (WBCs and heterophils), respiratory burst, lysozyme activity, and C-reactive protein were also affected in *O. niloticus* exposed to diazinon [66].

There is evidence that the complement system, lysozyme activity, respiratory burst, and peroxidase activity are dysregulated by OPs [67]. Chlorpyrifos exposure induced a reduction of lysozyme, respiratory burst, myeloperoxidase, phagocytosis, and complement system in *Pseudetroplus maculatus* Bloch [68]. In carp (*C. carpio*) exposed to trichlorfon, some parameters of nonspecific immunity, such as the phagocytic ability of neutrophils, phagocytic index, lysozyme, and PMNCs respiratory burst, were decreased [69]. Moreover, after exposure to dichlorvos, innate parameters (ceruloplasmin, lysozyme, hemagglutinins) were slightly affected in *C. carpio* [70].

Some contradictory effects were reported on lysozyme activity, which increased significantly in the liver and spleen exposed acutely to diazinon. However, during subacute and sub-chronic exposure, lysozyme activity decreased in the plasma, liver, kidney, and spleen of beluga (*Huso huso* L.) [71]. This phenomenon was also reported on Nile tilapia (*O. niloticus*) exposed to chlorpyrifos which triggered an increase in lysozyme activity in plasma; however, lower pesticide concentrations did not cause any effect on the enzymatic activity [72]. Diazinon also stimulates some non-specific immune defense mechanisms of grass carp (*Ctenopharyngodon idella* Valenciennes) by enhancing lysozyme activity [73]. Differential effect by OP exposure was also reported in common carp (*C. carpio*) exposed to chlorpyrifos, where lysozyme activity at the earlier stages of exposure was unaltered, but was inhibited at the late stages in serum, this same effect was observed in C3 mRNA [74]. Thus, in the case of contaminant-induced immunosuppression, there would be an increase in the susceptibility to Gram-positive bacteria and other pathogenic microorganisms [72].

Other alterations to immune mechanisms by OPs exposure are phagocytic parameters. In line with this, malathion has a negative effect on the innate immune response (WBC, ROS production, and phagocytic activity) in *C. carpio carpio* [75]. Additionally, acute and chronic exposure to chlorpyrifos effects leukocyte phagocytic capacity [76,77,78]. Furthermore, malathion reduces the phagocytic capacity of Murray codfish (*Maccullochella peelii* T. L. Mitchell) [79]. In addition, phagocytosis was reduced, and an ROS production increment was reported in *O. niloticus* exposed to diazinon [28].

Several cytotoxic alterations have been reported in Nile tilapia (*O. niloticus*) exposed to diazinon: this pesticide negatively affected the intracellular calcium flux, ERK1/2 phosphorylation (pERK1/2), and mitochondrial membrane potential (ΔΨm), while increased apoptosis and senescence were observed in spleen mononuclear cells (SMNC) [29].

#### 3.2.2. Amphibia

Immunosuppressive effects, such as an enzyme activity levels decrement, have been demonstrated in populations of *Leptodactylus latrans* and *Hyloxalus pulchellus* [80]. In terms of cell counts, a drastic reduction in WBC content was observed in *Bufo melanostictus* Schneider (Common Indian toad) exposed to malathion [81]. In *Rana pipiens* Schreber, a significant decrease in splenocyte numbers (cellularity), and phagocytic activity was observed in frogs sampled at pesticide-impacted sites [82]. In *Rana temporaria*, a lower number of blood leukocytes was also observed when they were exposed to high concentrations of tetrachlorvinphos compared to unexposed animals [83].

#### 3.2.3. Reptilia

Studies on the effect of OPs on the mechanisms of innate immunity in reptiles are scarce; the reports published to date are presented below. On glyphosate-based formulation exposition of *Caiman latirostris*, a decrease in WBC counts, as well as a higher percentage of heterophils, was reported [84]. In the Caspian pond turtle (*Mauremys caspica caspica* Gmelin) exposed to diazinon, a reduction in serum complement, lysozyme activity, and phagocytosis were reported, while the heterophil/lymphocyte ratio increased [85]. Moreover, in broad-snouted caiman (*Caiman latirostris* Daudin), a lower complement system activity was reported after commercial-mixed glyphosate exposure [86]. A multiple exposure assay of glyphosate, chlorpyrifos, and cypermethrin, during embryo development of tegu lizard (*Salvator merianae* Duméril and Bibron) detected a decrease in heterophils and the heterophil/lymphocyte ratio, while natural antibody titers increased [87].

#### 3.2.4. Birds

Birds are non-target species and OPs exert immunotoxicity. Methidathion and chlorpyrifos exposure in young chickens reduces WBC and neutrophil count [88]. Moreover, in broiler chicks, exposure to monocrotophos reduces active splenic macrophages [89]. Chlorpyrifos administered to broiler chicks caused a dose-dependent decrease in phagocytic activity [90]; while no difference in the innate immune response in sub-chronic exposure to malathion of birds (*Coturnix coturnix japonica* Temminck and Schlegel) was reported [91].

In ovo exposure assays to OPs also induce immunosuppressive effects, in line with this, on chicks that were exposed in ovo to a pesticide mixture (chlorpyrifos 50%; cypermethrin 5% and spinosad 45%), a decrement on phagocytic activity was observed [92]. Chlorpyrifos and cypermethrin insecticides in ovo exposure in domestic hens (*Gallus gallus domesticus* Brisson) increased eosinophils and decreased monocytes in the F1 generation, while in F2, both cell populations decreased [93].

#### 3.2.5. Mammals

The toxic effects of OPs exposure on the immune response have mainly focused on mammals, using rat, mouse, rabbit, and human models. The main findings reported in animal models are mentioned below.

In mice, rats, and rabbits, alteration in macrophage cell migration has been observed after exposure to malathion [94], and similar effects were reported in albino rats sub-chronically exposed to phosphamidon [95]. Additionally, in rats, in vitro exposure to malathion directly induced a decrease in nitrite production, and LPS-induced macrophages decrease TNF-α release [96]. Likewise, in rats chronically exposed to acephate, TNF-α, and iNOS levels were lower in LPS-induced macrophages [97]. OPs are also linked to mast cell/basophil dysregulation, which can be associated with allergies. In this context, malathion exacerbates mast cell degranulation and phagocytosis in mice [98].

Perinatal exposure to methamidophos interferes with neutrophil recruitment through IL-6 and interferon in response to the respiratory syncytial virus (RSV) in the lung tissue in the offspring generation of mice [99]. In addition, 2,2-Dichlorovinyl dimethyl phosphate (DDVP) induced inhibition of NK, limphokine-activated killer cells (LAK), and Cytotoxic T lymphocytes (CTL) activities in KO mice, through the impairment of the FasL/Fas pathway-associated phenomenon [100].

In humans, multiple exposures to immunomodulatory pollutants make it difficult to assess the particular OPs immuno-toxic effects. However, taking together all scientific pieces of evidence indicates that OPs exert immuno-toxic effects. In this context, neutrophil-mediated immunity can be altered by OPs, as reported in workers occupationally exposed [101]. Besides, metabolites of the pesticide malathion, have been shown to induce histamine release in human basophilic cells [102].

The complement system, a significant line of defense against infections, can also be altered by OPs exposure [103]. Dimethoate and chlorpyrifos dysregulated pro-inflammatory cytokines (IL-1β and IL-8), while, the anti-inflammatory cytokine (IL-10), as well as Akt and ERK, were downregulated in dendritic cells from OPs exposure [104]. IFN-β production by macrophages was inhibited by malathion exposure [105].

### 3.3. OP-Mediated Modulation of the Adaptive Immune Response in Vertebrates

#### 3.3.1. Fish

The parameters of adaptive immunity and their alteration in fish due to exposure to OPs have been little studied. In this regard, [106] reported that in vitro exposure to diazinon caused a decrease in plasma IgM in Nile tilapia (*O. niloticus*). Similarly, in carp (*C. carpio*) exposed to glyphosate-POEA, IgM mRNA levels were lower than in the control group [107]. In a study conducted on *C. carpio* exposed to chlorpyrifos, IgM concentration decreased in the serum and spleen, but not in the kidney [74]. This may be related to the results published by [108], which reported a decrease in serum levels of antibodies and antibody-forming cells (AFC) in tilapia exposed to edifenphos and glyphosate. Similarly, a decrease in lymphocytes was reported in rainbow trout *Oncorhynchus mykiss* exposed to diazinon [64]. A dose-dependent proliferation suppressive effect was also observed in *C. carpio* lymphocytes isolated from the pronephros (primary hematopoietic lymphoid organ) exposed in vitro to trichlorfon or dichlorvos [70].

#### 3.3.2. Amphibia

Given that amphibians are frequently exposed to agricultural pesticides, it is possible that these pollutants alter their immune system and render them more susceptible to different pathogens [60]. In the specific case of OPs, exposure to chlorpyrifos in toad tadpoles (*Odontophrynus carvalhoi*) altered the WBC, which could alter the ability of tadpoles to respond to environmental stress, make them more susceptible to infection by various pathogens, and thus reduce their chances of survival [109].

#### 3.3.3. Reptilia

To date, no studies have been reported evaluating the effect of OPs on the adaptive immune response in reptiles. Therefore, efforts should be made to evaluate the alteration of these parameters. The only published precedent is related to environmental monitoring, where adaptive parameters were evaluated in reptiles living in an area with agricultural activity and exposed to mixtures of pesticides (not defined), but the adverse effect on thymic T-cell maturation was demonstrated, suggesting an alteration in adaptive response mechanisms [110].

#### 3.3.4. Birds

It has been described that pesticides may cause negative effects and alterations in non-target species. In particular, the effect of quinalphos on humoral immune response in chickens was tested. In this study, all chicks were vaccinated with the Ranikhet disease vaccine on day 4 and Infectious bursal disease (IBD) on day 15. In addition, a group of chicks was given quinalphos in feed. There was a significant suppression in serum globulin, gamma globulin, and specific antibody titre against Ranikhet disease and IBD. B-lymphocyte blastogenesis was found to be reduced in chicks fed quinalphos in comparison to controls, indicating an immunosuppressive effect [111].

Birds in agricultural environments are commonly exposed to insecticides, mainly through the ingestion of invertebrates after insecticide application. A study on the Japanese quail (*Coturnix japonica*) as an avian model was carried out to determine short-term microbial community responses to a single dose of trichlorfon at low concentration in three sample origins of the gastrointestinal tract (GIT): caecum, large intestine, and feces. The study showed that ingestion of insecticide caused significant changes in the GIT microbiome. This study demonstrated the significant impact that OPs have on the avian gut microbiota, showing that a single small dose of trichlorfon caused dysbiosis in the GIT of the Japanese quail [112].

#### 3.3.5. Mammals

In mammals, several studies have been reported on OPs exposure and its effect on adaptive immune mechanisms, focusing on humoral parameters such as serum levels of antibodies, inflammatory cytokines, and lymphocyte proliferation and differentiation parameters. For the study of adaptive parameters, studies have focused on the use of model organisms (murinae and leporidae) and primary cell cultures. This is due to the complications in assessing the direct effect on humans due to the multiple contaminants to which they may be exposed. Therefore, some of the published reports have been conducted in case studies or in occupationally exposed populations and the reported results are described below.

In a study of rats exposed to the pesticide dimethoate, a decrease in the content of IgM plaque-forming cells and a delayed-type hypersensitivity reaction were observed [113]. On the other hand, a significant decrease in macrophage activity, serum lysozyme activity, and IL-2 and IL-6 levels, as well as oxidative stress and DNA damage in lymphocytes were observed in rats exposed to chlorpyrifos [114].

In addition, the effect of different OPs on cell populations has been described. In this regard, decreased antibody-dependent activity of neutrophils and NK cells was observed in rats after exposure to malathion and parathion [115]. Similarly, the effect of diazinon was studied in albino rats, which decreased the proliferation of blood mononuclear cells and blood T-cell subtypes (CD4+ and CD8+), as well as the reduction of total serum immunoglobulin and hemagglutination titers [116].

Exposure to OPs has been described to deregulate both B-cell maturation and T-cell differentiation in mammals. In this regard, IgM concentration, cytotoxic T lymphocyte count, IFNγ, and TNF-α production were decreased in mice exposed to parathion. In addition, Th2 cytokine production and GATA-3 gene expression were significantly increased [117]. Alongside that, increased expression of surface T-cell receptors and levels of Th1 cytokines (IFN-γ, TNF-α) and exacerbation of T-lymphocyte-mediated allergic reactions were induced in rats [118]. In rats exposed to fenitrothion, serum levels of TNF-α and IL-2 were increased, while it caused a reduction of IgG and IgM [119].

Specifically, for pets and livestock, OPs are commonly used to control pests and vector diseases, such as arthropods. However, these compounds are associated with acute intoxication and death [120]. Pets (cats and dogs) are mainly affected by OP intoxication (86.9%), while in other animals, such as livestock (horses, cows, lambs, and goats), these events occur sporadically [121]. In particular, OP-mediated immunosuppression has been reported in lambs, where oral exposure to monocrotophos led to lymphocyte decrement [122].

As previously mentioned, research focused on human exposure to OPs has been conducted in primary cell cultures. A recent study reviewed the immunotoxicity of pesticides that are currently used and those prohibited, focusing on T cells, B cells, NK cells, and macrophage alterations. Pesticides have several toxicological modes of action to induce mitochondrial dysfunction, endoplasmic reticulum (ER) stress, apoptosis, and cell cycle arrest [123].

Another study reported the effect of chronic intoxication of OPs when measured on the phagocytic activity of neutrophils. In addition, cell functions related to the immune system were evaluated, including the blood levels of pro-inflammatory cytokines TNF-α, IL-1β, and IL-6, and the cholinergic anti-inflammatory pathway activation. In this study, exposure to malathion and parathion methyl led to a decrease in the phagocytic activity of neutrophils, the activity of NK cells, and antibody-dependent cellular cytotoxicity. In addition, the function of the phagocytic-monocytic system after chronic poisoning of OPs compounds decreases, which is manifested by a reduction in the blood concentration of proinflammatory cytokines TNF-α, IL-1β, and IL-6. Further, chronic intoxication with OPs leads to the realization of the cholinergic anti-inflammatory pathway [115]. In human CD4+ T cells exposed to diethyl dithiophosphate (DEDTP), reduced T-cell proliferation and intracellular secretion of IL-2, IL-10, and IFN-γ were observed [19].

## 4. Conclusions

The immuno-toxic effects mediated by OPs affect both invertebrate and vertebrate organisms, with direct repercussions on the innate and adaptive immunity of exposed organisms. Acute exposure has been reported to cause impairments in phagocytosis, respiratory burst, ROS release, hemocyte/leukocyte cellular response, antibody production, cell proliferation, and cytokine release.

Hence, exposure to OPs can cause alterations in the various cells of the immune system, which can result in increased susceptibility to infections caused by opportunistic microorganisms, including viruses, bacteria, parasites, and fungi, thus necessitating studies in which the exposure to OPs in relation to an antigenic challenge is evaluated, causing an imbalance in the environment and in the health of organisms. In addition, effects related to environmental concentrations and pesticide mixtures should be considered. In addition, the range of species studied should be broadened, with a focus on non-target species (wildlife), and the implications of ecosystem effects (food webs).

## Data Availability

Not applicable.
